# Identification of transcription factor and microRNA binding sites in responsible to fetal alcohol syndrome

**DOI:** 10.1186/1471-2164-9-S1-S19

**Published:** 2008-03-20

**Authors:** Guohua Wang, Xin Wang, Yadong Wang, Jack Y Yang, Lang Li, Kenneth P Nephew, Howard J Edenberg, Feng C Zhou, Yunlong Liu

**Affiliations:** 1 Division of Biostatistics Department of Medicine, Indiana University School of Medicine, Indianapolis, IN 46202, USA; 2 Center for Computational Biology and Bioinformatics, Indiana University School of Medicine, Indianapolis, IN 46202, USA; 3 School of Computer Science and Technology, Harbin Institute of Technology, Harbin, Heilongjiang, 150001, China; 4 College of Automation, Harbin Engineering University, Harbin, Heilongjiang 150001, China; 5 Medical Sciences, Indiana University School of Medicine, Bloomington, IN 47405, USA; 6 Department of Biochemistry and Molecular Biology, Indiana University School of Medicine, Indianapolis, IN 46202, USA; 7 Center for Medical Genomics, Indiana University School of Medicine, Indianapolis, IN 46202, USA; 8 Department of Anatomy and Cell Biology, Indiana University School of Medicine, Indianapolis, IN 46202, USA

## Abstract

This is a first report, using our *MotifModeler* informatics program, to simultaneously identify transcription factor (TF) and microRNA (miRNA) binding sites from gene expression microarray data. Based on the assumption that gene expression is controlled by combinatorial effects of transcription factors binding in the 5'-upstream regulatory region and miRNAs binding in the 3'-untranslated region (3'-UTR), we developed a model for (1) predicting the most influential *cis*-acting elements under a given biological condition, and (2) estimating the effects of those elements on gene expression levels. The regulatory regions, TF and miRNA, which mediate the differential genes expression in fetal alcohol syndrome were unknown; microarray data from alcohol exposure paradigm was used. The model predicted strong inhibitory effects of 5' *cis*-acting elements and stimulatory effects of 3'-UTR under alcohol treatment. Current predictive model derived a key hypothesis for the first time a novel role of miRNAs in gene expression changes associated with abnormal mouse embryo development after alcohol exposure. This suggests that disturbance of miRNA functions may contribute to the alcohol-induced developmental deficiencies.

## Introduction

Understanding global transcriptional and post-transcriptional regulatory mechanisms is a fundamental goal of the post-genomic era [1]. We have previously developed a model-based procedure, *MotifModeler*, to predict *cis*-acting elements in the promoter region that affect gene regulation [2]. Here we report a novel extension of this approach to simultaneously identify potential transcription factor and microRNA (miRNA) binding sites from microarray-derived gene expression data and genomic DNA sequences. Conventional computational methods using microarray data to investigate transcriptional regulation focus mainly on identification of transcription factor binding sites [3-5]. However, many cellular processes contribute to changes in eukaryotic gene expression levels, such as the combination of transcriptional events and mRNA degradation (triggered, for example, by miRNAs, or RNA binding proteins). Thus, computational approaches are needed to integrate such cellular processes. In the present study, we describe a novel model-based approach for identifying potential transcription factor and miRNA binding sites from microarray-derived gene expression data and genomic DNA sequences.

MiRNAs bind to complementary sites on the 3'-untranslated regions (3'-UTR) of target mRNAs to induce cleavage and repression of translation [6-8]. In the past decade, several hundred miRNAs have been identified in mammalian cells [9,10]. Accumulating evidence indicates that miRNAs play critical roles in multiple biological processes, including cell cycle control, cell growth and differentiation, apoptosis, and embryo development [6,11-15]. At the biochemical level, miRNAs regulate mRNA degradation in a combinatorial manner, *i.e*., individual miRNA regulates degradation of multiple genes, and regulation of a single gene may be conducted by multiple miRNAs [16,17]. This combinatorial regulation is thought to be similar in scope to transcription factor partner regulation [6]. Therefore, in addition to transcription factors and other DNA/RNA-binding proteins, comprehensive investigations into transcriptional mechanisms underlying alterations in global gene expression patterns should also consider the emerging role of miRNAs [18].

We previously developed a model-based procedure, *MotifModeler *[2], for identifying *cis*-acting elements involved in particular biological processes. By testing random subsets of all possible motifs of a fixed size in the 5'-regulatory region and selecting those motifs that best fit a combinatorial model of gene expression levels, *MotifModeler* identified many known transcription factor binding sites along with novel binding sites. In the current study, we extend the *MotifModeler* algorithm to include the repressive effects of *cis*-acting elements in the 3'-untranslated region. The *cis*-acting elements in the 3'-untranslated region could represent either miRNA binding sites or protein binding sites.

A gene expression profile in an established fetal alcohol syndrome (FAS) biological model system was used to demonstrate the new model. A whole embryo culture system was used to examine the effects of alcohol on embryos without the complications of maternal factors [19]. This* in vitro* model also provided a difined alcohol concentration in strictly controlled embryonic stage, thus allow for a stringent comparison in high throughput analysis. The gene expression was analysed in parallel to the developmental deficit [20]. The genome-wide gene expression profiles determined on Affymetrix microarrays were analysed to detect potential *cis*-acting elements which may underlie the effects of alcohol.

## Methods

### Biological model system

To study gene expression changes associated with fetal alcohol exposure, we utilized global gene expression profiles from experiments performed by our group [20]. We have previously defined the timing, concentration, and pattern of alcohol exposure, and we recently characterized gene expression patterns in parallel with developmental abnormalities [20]. In the previous investigation, beginning on gestational day 8.25 (E8.25), 4 C57BL/6 mouse embryos were treated with alcohol (peaking at 88mM) and 4 control without alcohol for 46 hours, and total RNA was isolated for expressional microarray analysis using the Affymetrix Mouse Genome 430 2.0 GeneChip®. In this study, we focused on microarray data that measure the global gene expression profiles in the whole mouse embryos, comparing control embryos (with closed neural tubes) with alcohol treated embryos that had phenotype containing open neural tubes (4-array by 4-array comparison, GEO Accession# GSE9545). After removing genes that were not reliably detected in at least one of the two conditions (control vs. ethanol treated), we selected the 528 probe sets, the expression levels of which were altered significantly (*p* < 0.05, and fold change > ±1.5). Probe sets not reliably detected [21] and with absent annotation were removed; for genes with multiple probe sets, we selected the one with the largest fold change to represent the gene. This left 269 genes for analysis, in which 94 were up-regulated and 175 were down-regulated by ethanol.

### Regulatory sequences

For the 269 differentially expressed genes, regulatory sequences in both the 5'-proximal flanking region (up to 1,000-bp in length) and the annotated 3'-untranslated region were retrieved from the Build 35 assembly by NCBI (National Center for Biotechnology Information) and downloaded from the UCSC genome browser [22]. Potential transcription factor binding sites and miRNA binding sites were selected from all 6- and 7-bp DNA elements, respectively. After combining reverse complementary sites, there were 2,080 6-bp motif candidates in the 5'-proximal regulatory region. In the 3'-untranslated region, complementary sites were not combined since miRNAs bind to the single-stranded RNA; thus there is a pool of 16,384 7-bp motif candidates.

### Modelling gene expression data

A simplified quantitative relationship between gene expression levels and transcription factor and miRNA binding sites can be formulated as an extension of *MotifModeler *[2]:

$$g_{k}=\sum_{i\in T_{k}}t_{k,i}x_{i}-\sum_{j\in M_{k}}m_{k,j}y_{j}$$

where,

• *g_k_* logarithmic ratio of mRNA expression levels of the *k*-th gene in the treatment group comparing to control;

• *t_k,i_* number of transcription factor binding site *i* in the 5'-regulatory region of the *k*-th gene;

• *T_k_* all the transcription factor binding sites having occurrences in the 5'-regulatory region of the *k*-th gene;

• *x_i_* functional levels of the *i*-th transcription factor binding site;

• *m_k,j_* number of miRNA binding site *j* in the 3'-UTR of the *k*-th gene;

• *M_k_* all the miRNA binding sites having occurrences in the 3'-UTR of the *k*-th gene;

• *y_j_* functional levels of the *j*-th miRNA binding site;

The biological implication of this equation is that the measured gene expression level *g_k_* is modeled by a combinatorial effect of transcription, controlled by 5' *cis*-acting elements, and degradation, regulated by miRNAs and their binding sites in the 3'-untranslated region. Note, however, that the equation can accommodate both positive and negative effects of any site.

In Eq. 1, the functional levels of each transcription factor binding site (*x_i_*) or miRNA binding site (*y_j_*) can be estimated using a least-squares approach. Eq. 1 can be rewritten in a matrix formulation:

$$\underline{G}=T\underline{X}-M\underline{Y}=\left[\begin{array}{cc} T & M \end{array}\right]•\left[\begin{array}{c} \underline{X}\\ -\underline{Y} \end{array}\right]$$

where *T* = (*t_k,i_*) and *M* = (*m_k,j_*); If we define *N* = [*T*, *M*] and *A* = [*X*^T^, -*Y*^T^]^T^, the *X* and *Y* can then be estimated:

$$\underline{\hat{A}}={\left(N^{T}N\right)}^{-1}N^{T}\underline{G}$$

The first *n_t_* and the last *n_m_* elements in *A* vector can be referred as the estimation for *X* and *Y*, respectively. Meanwhile, the model error based on a given selection of transcription factor and miRNA binding sites will be defined as the sum square of the differences between observed and predicted mRNA expression levels:

$$E=\sum_{k=1}^{n}{\left(g_{k}-\left(\sum_{i\in T_{k}}t_{k,i}{\hat{x}}_{i}-\sum_{j\in M_{k}}m_{k,j}{\hat{y}}_{j}\right)\right)}^{2}$$

where *n* is total number of genes.

As in *MotifModeler*[2], the new motif selection procedure evaluates the effects of all potential transcription factor and miRNA binding sites in the 5'-regulatory sequences and 3'-UTR. The procedure was completed in *N_p_*=200,000,000 iterations. In each iteration, *n_t_* =15 and *n_m_* =15 motif candidates were randomly selected from their respective pools of candidates. Model errors based on each set of motifs were calculated based on Eq. 4. Since a smaller model error implies a more influential binding site, a transcriptional contribution score (TCS) was assigned to each selected candidate in the set according to the following formulation:

$$TCS_{i}=\sum_{c\in C_{n_{t,},n_{m},i}}\frac{1}{E_{c}^{\alpha }}$$

where *E* is the model error derived in Eq. 5; $C_{n_{t,},n_{m},i}$ represents all the combinatorial selections (having *n_t_* and *n_m_* transcription factor binding sites (TFBS) and miRNA binding sites (MBS), respectively) that include the *i*-th TFBS or MBS. It has to be noted that the predicted binding sites are indeed *cis*-acting elements (CAE), which is assumed as TFBS and MBS, respectively. In Eq. 5, **α** is the power factor that influences the effect of single selections (α > 1). In each iteration, a larger α value usually amplifies the additive contribution of the motif sets with smaller model errors (here, we used *α* = 5). Similarly, cumulative functional levels of each TFBS and MBS were calculated:

$X_{i}=\sum_{c\in C_{n_{t,},n_{m},i}}{\hat{x}}_{c}$ and $Y_{j}=\sum_{c\in C_{n_{t,},n_{m},j}}{\hat{y}}_{c}$

where $\hat{x}$ and $\hat{y}$ are the estimated functional levels of TFBS and MBS in each iteration (Eq 3.). Overall, the proposed motif selection procedure can be summarized as:

1. Randomly pick *n_t_* and *n_m_* elements from pools of TFBS and MBS.

2. Calculate the predicted model error E (Eq. 4).

3. Calculate the current contribution score (TCS) of each TFBS and MBS candidate as reciprocal to E, and their and functional levels (*X* and *Y*).

4. Add the current contribution score to the cumulative transcriptional contribution score (TCS) and functional levels (*X* and *Y*) (Eq. 6).

Repeat the procedure (1-4) N_rep_ times. Usually, N_rep_ will be selected so that each CAE and MBS candidate is evaluated 10,000 times. All the customized programs were written using R 2.4.0 ().

### Permutation analysis

In order to evaluate the significance of the calculated TCS scores, permutation analysis was implemented by randomizing the orders of gene expression data (G in Eq. 2). This permutation not only effectively disconnects the functional relationships of genes and their promoter sequences, but also preserves the promoter contents and the distribution of gene expression.

### Correlation of predicted motifs with biologically-known TFBS and miRNA binding sites

We evaluated the sequence similarities of the 5' *cis*-acting elements (putative TFBS) to 774 distribution matrices of aligned binding sites in the TRANSFAC database using position-specific scoring matrices (PSSM) [2]. For the predicted miRNA binding sites, we compared the selected 7-bp motifs with experimentally-verified miRNAs in the miRNA registry [9], searching against the complementary sequences of 375 mouse miRNAs.

## Results

### Selection of TFBS and MBS

**Figure 1:**
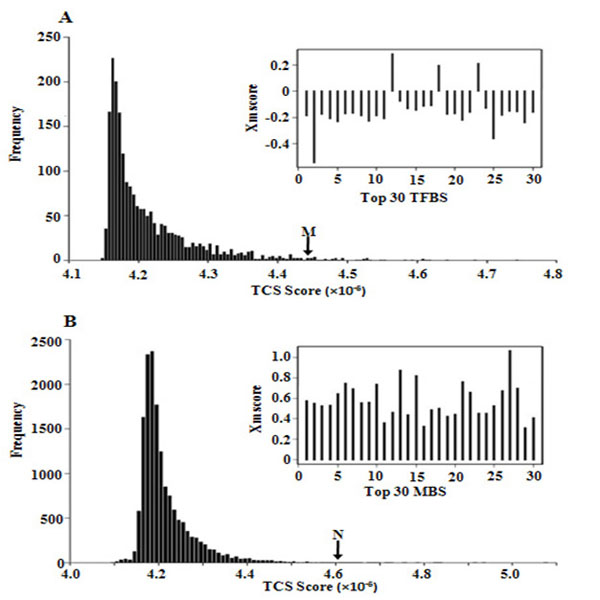
**TCS and Xm scores for 5'-regulatory region (promoter) and 3'-untranslated region**. (A) Histogram of TCS scores of the 2,080 6-bp motifs, and predicted functional levels (Xm) of top 30 motif candidates. (B) Histogram of TCS scores of the 15,870 7-bp motifs, and predicted functional levels (Xm) of top 30 motif candidates.

Potential TFBS and MBS were selected based on the number of occurrences of 6-bp elements in 5'-proximal flanking region of transcription starting sites and 7-bp elements in 3'-untranslated regions of mRNA, respectively, as detailed in the methods. The histograms of TCS scores of 6-bp TFBS candidates and 7-bp MBS candidates are shown in Fig. 1. Note that most of 6-bp and 7-bp candidates are not real binding motifs. Only the ones with largest TCS scores made significant contribution to the alteration of gene expression after alcohol treatment.

We selected the 30 TFBS candidates (6-bp motifs) and 30 MBS candidates (7-bp motifs) with the highest TCS scores. Permutation analysis suggested that the false discovery rate for the top 30 TFBS and MBS candidates are 3.8% and 2.6%, respectively. The *Xm* values for the 30 TFBS and MBS candidates that received the highest TCS scores are shown in Fig. 1 and Table 1. In the 5'-proximal regulatory region, 27 out 30 predicted 6-bp motifs received negative *Xm* values. This implies decreased capability of the 5'-end promoters in initiating transcription after treatment with alcohol. Strikingly, the *Xm* values of all the top 30 7-bp motifs selected in the 3'-UTR were positive. Considering the fact that miRNAs trigger sequence-specific RNA degradation by binding in the 3'-UTR, a positive *Xm* values are indicative of decreased capabilities of a miRNA to trigger RNA degradation in the presence of alcohol.

**Table 1:** Transcriptional contribution scores (TCS) and estimated functional levels (*Xm*) of top 30 selected TFBS and MBS

**5'-proximal regulatory region**				**3'-untranslated region**		
TFBS	TCS(×10^-6^)	*Xm*		MBS	TCS(×10^-6^)	*Xm*
CTCCAA	4.744	-0.186		TGGTGTC	5.073	0.576
GCGTTA	4.684	-0.546		GCTGTGC	4.936	0.550
CACATA	4.640	-0.175		CTGGTGT	4.914	0.526
AACTGA	4.615	-0.207		TGACCAG	4.830	0.531
CCTACC	4.613	-0.232		CTCCCAA	4.824	0.642
GACACA	4.608	-0.171		GTGTTGC	4.782	0.747
AACACA	4.606	-0.168		TATGTAG	4.756	0.694
CACAAC	4.595	-0.185		ACCTGGC	4.724	0.554
ACAAGT	4.582	-0.229		TGGAGCA	4.724	0.560
CCACAA	4.552	-0.187		ACAACAG	4.713	0.735
AAGTTA	4.548	-0.207		GCTGTTT	4.698	0.359
GCATGC	4.533	0.285		AAGACAA	4.692	0.460
ACACAC	4.530	-0.074		TGTCTCG	4.687	0.872
AGGGGA	4.527	-0.134		GTGCCTC	4.687	0.438
CAAGGG	4.525	-0.146		TTGAGGT	4.687	0.821
AAAATA	4.523	-0.113		CTGTGCT	4.676	0.322
CAGCCC	4.522	-0.111		GCTCCCT	4.671	0.484
ATCTTG	4.519	0.195		GGTCCTG	4.655	0.499
AAGTAA	4.512	-0.176		CAGGACT	4.655	0.421
ACGCCC	4.494	-0.171		GGGGCCA	4.655	0.442
CTCCGA	4.492	-0.221		AGGGAAC	4.645	0.759
TCACAA	4.491	-0.158		GTGTATC	4.639	0.658
GCCGGC	4.487	0.213		TTCTAGA	4.639	0.450
AGGGCA	4.484	-0.130		GTGCTCA	4.639	0.449
ACGCTA	4.484	-0.362		GGTGTCT	4.629	0.526
AAGCAC	4.484	-0.184		AGACAAC	4.613	0.673
AGGACT	4.479	-0.152		GAATTGC	4.613	1.065
CTGCAG	4.477	-0.156		GGTACTG	4.613	0.694
CCCATA	4.473	-0.238		CTCTTCC	4.608	0.311
GGCACA	4.469	-0.159		ACAGCCT	4.602	0.407

### Permutation analysis

**Figure 2:**
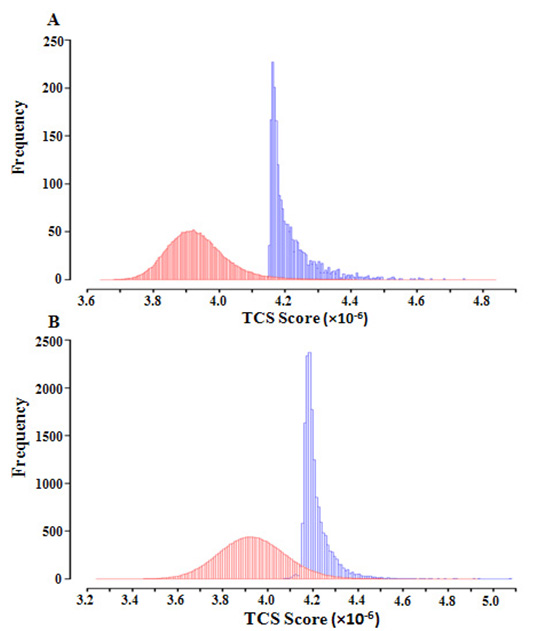
**Permutation analysis**. Histograms of TCS scores of (A) 6-bp motifs in the 5'-proximal regulatory region; and (B) 7-bp motifs in the 3'-untranslated regions. TCS scores were calculated based on gene expression levels with randomized orders (red histograms) and original orders (blue histograms).

Histograms of predicted TCS scores based on experimentally-determined gene expression data and randomized gene expression data are shown in Fig. 2. We observe significant lower TCS scores for the randomized gene expression data in both 5'-proximal regulatory regions and 3'-untranslated regions.

### Correspondences of predicted binding sites to known binding sites

We compared the top 30 predicted 6-bp motifs in the promoter region (potential TFBS in Table 1) with 741 biologically-known binding sites in the TRANSFAC database. Of these, significant sequence similarity was observed for 20 TFBS with at least two of top 27 predicted 6-bp motifs (Table 1) showing inhibitory effects after alcohol treatment (*Xm*<0, Table 2). These factors included serum response factor, GC box elements, early growth response gene, stress-response element, and stimulating protein 1 known to be the group of stress related early-expressed genes (Table 2). Similarly, three TFBS had sequence similarity with at least 2 of the top 3 predicted 6-bp motifs showed stimulatory effects after alcohol treatment (*Xm*>0, Table 3). These factors included *Evi-1*, a murine myeloid leukaemia-associated transcription factor, tumor suppressor p53, and nuclear respiratory factor 1 (*Nrf-1*) which related to energy consumption and apoptosis.

**Table 2:** Correspondence of 27 negative TFBS to the TRANSFAC database

ID*	**Score**	**TFBS**	**# of matches**	**Name**
M00152	13.86	SRF	3(14/18)	Serum response factor
M00255	13.62	GC box	3(13/14)	GC box elements
M00245	13.32	Egr-3	2(12/12)	Early growth response gene 3 product
M00246	12.65	Egr-2	2(12/12)	Egr-2/Krox-20 early growth response gene product
M00982	12.55	KROX	3(12/14)	
M00731	12.98	Osf2	3(7/8)	Runt-related transcription factor 2 (Runx2)
M00154	12.15	STRE	2(8/8)	Stress-response element
M00933	12.07	Sp-1	3(8/10)	Stimulating protein 1
M00932	10.55	Sp-1	3(8/13)	Stimulating protein 1
M00931	8.83	Sp-1	2(6/10)	Stimulating protein 1
M00008	8.47	Sp1	2(6/10)	Stimulating protein 1
M00456	11.26	FAC1	3(10/14)	Fetal Alz-50 clone 1
M00360	10.29	Pax-3	3(9/13)	Pax-3 binding sites
M00134	10.19	HNF-4	3(13/19)	Hepatic nuclear factor 4
M00411	9.68	HNF4α1	2(12/15)	Hepatocyte nuclear factor 4
M00316	10.04	Hogness BOX	3(15/30)	Imperfect Hogness/Goldberg Box
M00467	9.83	Roaz	2(8/14)	Rat Olf-1/EBF-associated zinc finger protein
M00977	9.78	EBF	2(7/11)	Early B-cell factor
M00119	9.28	Max	2(12/14)	Max
M00118	9.20	c-Myc:Max	2(12/14)	c-Myc:Max heterodimer
M00953	9.13	AR	3(13/27)	High affinity binding sites for androgen receptor
M00332	8.78	Whn	2(6/11)	Winged-helix factor nude
M00330	8.64	Major T-antigen	2(12/19)	Major T-antigen binding sites
M00763	8.37	PPAR	2(9/13)	Peroxisome proliferator-activated receptor
M00778	8.33	AhR	2(6/11)	Aryl hydrocarbon / dioxin receptor
M00769	8.12	AML	2(7/15)	Leukemia
M00256	8.11	NRSF	2(12/21)	Neuron-restrictive silencer factor

**Table 3:** Correspondences of 3 positive TFBS to the TRANSFAC database

ID*	**Score**	**TFBS**	**# of matches**	**Name**
M00078	9.49	*Evi-1*	2 (11/16)	Ectopic viral integration site 1 encoded factor
M00034	9.15	*p53*	2 (12/20)	Tumor suppressor p53
M00652	7.53	*Nrf-1*	1 (6/10)	Nuclear respiratory factor 1

We investigated the correspondences of the predicted 7-bp motifs with mature mouse miRNA sequences documented in the miRNA registry. Partial matches for sequences in 10 mature miRNAs were seen; furthermore, a perfect match for one of the top 30 predicted 7-bp motifs was observed (Table 4). Six of 10 of these had been previously reported in neuron and mouse embryo development (indicated by a ^†^ in Table 4). Mature miRNAs that matched at least two of top 30 predicted 7-mers (one G-U matched allowed) are listed in Table 5.

**Table 4:** MiRNAs that match perfectly with predicted 7-bp motifs.

miRNA	7-bp motif	MBS index	Match position	Mature miRNA sequence*
miR-489	TGGTGTC	1	4-10	aat**GACACCA**catatatggcagc
miR-666^†^	GCTGTGC	2	6-12	agcgg**GCACAGC**tgtgagagcc
miR-292-3p^†^	ACCTGGC	8	8-14	aagtgcc**GCCAGGT**tttgagtgt
miR-196b^†^	ACAACAG	10	12-18	taggtagtttc**CTGTTGT**tgg
miR-685^†^	GTGCCTC	14	15-21	tcaatggctgaggt**GAGGCAC**
miR-195	CTGTGCT	16	5-11	tagc**AGCACAG**aaatattggc
miR-330^†^	CTGTGCT	16	5-11	gcaa**AGCACAG**ggcctgcagaga
miR-424	GAATTGC	27	6-12	cagca**GCAATTC**atgttttgga
miR-219	GAATTGC	27	14-20	tgattgtccaaac**GCAATTC**t
miR-488*^†^	ACAGCCT	30	6-12	ttgaa**AGGCTGT**ttcttggtc

**Table 5:** MiRNAs that match with two predicted 7-bp motifs (one G-U pair allowed)

miRNA	7-bp motif	MBS index	Match position	Mature miRNA sequence*
miR-195	GTGTTGC	6	3-9	ta**GCAGCAC**agaaatattggc
	CTGTGCT	16	5-11	tagc**AGCACAG**aaatattggc
miR-196b	ACAACAG	10	12-18	taggtagtttc**CTGTTGT**tgg
	AGGGAAC	21	7-13	taggta**GTTTCCT**gttgttgg
miR-330^†^	CTGTGCT	16	5-11	gcaa**AGCACAG**ggcctgcagaga
	GGTCCTG	18	9-15	gcaaagca**CAGGGCC**tgcagaga
miR-666^†^	GCTGTGC	2	6-12	agcgg**GCACAGC**tgtgagagcc
	CTGTGCT	16	5-11	agcg**GGCACAG**ctgtgagagcc
miR-710^†^	CAGGACT	19	4-10	cca**AGTCTTG**gggagagttgag
	CTCTTCC	29	11-17	ccaagtcttg**GGGAGAG**ttgag

## Discussion

In this study, we extended the application of *MotifModeler* to simultaneously identify putative *cis*-acting elements that represent both transcription factor and miRNA binding sites from array-derived gene expression data. Using the microarray data of alcohol-induced gene expression in FAS mouse embryos with distinct biological consequence, our model predicted many TFBS and MBS whose functions differ as a result of alcohol treatment.

Remarkably, in this particular model, it is predicted that most of the 5' motifs showed down-regulatory effects, and all of the 3' miRNA motifs showed up-regulatory effects on gene expression after alcohol treatment. This is clearly shown in Fig. 1B, where most of predicted 6-bp motifs (27 out of top 30) in the 5'-end demonstrated inhibitory effects (*Xm* < 0) and all the top 30 predicted 7-bp motifs in the 3'-end demonstrated stimulatory effects (Xm > 0). This unbalance observation is unlikely to be random. There was a developmental delay caused by alcohol treatment, as shown by morphological analyses [20]. Therefore, one possible interpretation is that most genes that were at higher levels after alcohol treatment were highly expressed in a previous developmental stage and were not appropriately down-regulated. This could be due to delayed expression of miRNAs that would normally reduce expression.

A number of negative-TFBS are found closely related to the down-regulation of identified gene groups or function of the gene clusters related to developmental deficit in FAS. For example, the Egr-2/Krox-20 early growth response gene product, and early growth response 3 gene product (*Egr-2, Egr-3*) related to down regulation of Growth GO set related to growth deficit. The Krox, Pax3, and Winged-helix factor nude (Whn) homeodomain are fate-determinants for early development of neural axis and neural patterning. The Neuron-restrictive silencer factor (NRSF), Rat Olf-1/EBF-associated zinc finger protein (Roaz), and Runt-related transcription factor 2 (Osf2, a.k.a. Runx2) are mediating neural specification; these two TF groups together are plausible regulators for down-regulated neurodevelopmental genes seen in embryos with neurodevelopmental deficit [19,20]. Another outstanding set of predicted TFs, AML, Hogness BOX, and a murine myeloid leukaemia-associated transcription factor (Evi-1) are related to hemopoiesis. They may mediate the global decrease of hemopoiesis genes observed in the microarray data. The group of TFs, nuclear respiratory factor 1 (Nrf-1), tumor suppressor P53 (p53), max, and c-Myc, are predicted which are known to be closely related to mediating apoptosis. The up-regulation of Nrf-1 and P53, and down-regulation of Max-c-Myc may mediate to the downstream gene expression and apoptosis in the alcohol treated embryos. In summary, these predicted TFs / TFBS are closely related to the alteration of core gene expressions related to growth, neural specification, hemopoiesis, and apoptosis which are major developmental deficits in the FAS.

Based on several recent reports that regulatory targets of miRNAs can be identified by searching conserved complementarily motifs in the 3'-UTR, we selected 7-bp motifs as putative miRNA binding sites. Several recent studies suggested that the complementarity of 5'-extremity of miRNA and 3'-UTR of the target gene is critical for the miRNA function [16,23-25]. Further analysis indicated that starting from whole-genome alignments of five vertebrates, regulatory targets of miRNAs can be identified by searching conserved complementarity, on the 3'-UTR, to the 6-bp seed (nucleotides 2-7) of miRNA. An additional requirement of either conserved matches at nucleotide 8 of miRNA or conserved adenosines at position 1 of target mRNA greatly increases signal-to-noise ratios of the prediction, and therefore is desired to anchor the miRNA binding [18].

MiRNA target prediction is a challenging and unsettled area. Most bioinformatic procedures to predict miRNA targets take advantage of cross-species comparison, based on the assumption that target sites of miRNAs are evolutionarily conserved [10,26,27]. A more recent study demonstrated that putative miRNA binding sites that are not conserved across evolution also mediate repression [28]. This observation not only allows but also requires us to identify potential miRNA binding sites from genomic sequences of single organism, as implemented here. Other approaches have predicted post-transcriptional mechanisms using sequence information within one species [29,30]. Our method, however, is the first attempt to integrate the effects of TFBS and MBS into array-derived gene expression data analysis in a mammalian system.

As many RNA-binding proteins bind in the 3'-UTR, it is important to acknowledge that post-transcriptional regulation of gene expression is a multi-factorial phenomenon that includes miRNA and other molecules. In addition to facilitating translation, other factors can destabilize mRNA and thus serve similar functions as the RNA Induced Silencing Complex (RISC) recruited by miRNA [31]. An example of such an RNA-binding protein includes AU-rich elements (ARE) [32], a family of PUF proteins [33]. Our method identifies *cis*-acting elements in the 3'-untranslated region that could represent either miRNA binding sites or protein binding sites. This application in the field of fetal alcohol syndrome is in its infancy. Experimental evaluation is necessary to better understand the transcriptional and post-transcriptional mechanisms that regulate the global gene expression pattern.

## Competing interests

The authors declare that they have no competing interests.

## Authors' contributions

YL and FCZ contributed to the design of the study. GW and YL designed and performed the computational modeling and drafted the manuscript. XW, YW, JY, LL, KPN and HJE participated in coordination, discussions related to result interpretation and revision of the manuscript. All the authors read and approved the final manuscript.
